# The hypoxic ventilatory response and arousal burden predict the magnitude of ventilatory long‐term facilitation in humans with obstructive sleep apnoea

**DOI:** 10.1113/EP093163

**Published:** 2025-11-06

**Authors:** Jason H. Mateika, Danny Hammo, Dylan M. Kissane, Ali Azarbarzin

**Affiliations:** ^1^ John D. Dingell Veterans Affairs Medical Center Detroit Michigan USA; ^2^ Department of Physiology Wayne State University School of Medicine Detroit Michigan USA; ^3^ Department of Internal Medicine Wayne State University School of Medicine Detroit Michigan USA; ^4^ Division of Sleep and Circadian Disorders Brigham and Women's Hospital and Harvard Medical School Boston Massachusetts USA

**Keywords:** arousal, intermittent hypoxia, obstructive sleep apnoea, respiratory plasticity

## Abstract

The magnitude of progressive augmentation (PA) and ventilatory long‐term facilitation (vLTF) are two forms of respiratory plasticity that are enhanced in some humans with obstructive sleep apnoea (OSA). This response might be linked to repeated nocturnal exposure to intermittent hypoxia or other traits connected to OSA. A meta‐analysis was completed using data from 91 OSA participants who completed one of two mild intermittent hypoxia protocols during wakefulness. Two iterations of a subset regression analysis were completed to identify the best model that predicted the magnitude of PA or vLTF. Novel (e.g., arousal and hypoxic burden) or standard indicators of sleep apnoea (e.g., apnoea/hypopnoea index), anthropometric variables, protocol elements and physiological variables measured during wake and sleep were included as independent variables. After model selection, a multiple linear regression analysis was used to identify the most impactful variables in the model. The hypoxic ventilatory response (HVR) alone (*R* = 0.589, *P* < 0.001) or in combination with the arousal index (*R* = 0.625, *P* < 0.015 for both variables) predicted the magnitude of PA, whilst the HVR in combination with the arousal burden (*R* = 0.602, *P* < 0.001) or arousal index (*R* = 0.593, *P* < 0.002 for all variables) predicted the magnitude of vLTF. The HVR and markers of arousal are strong predictors of the magnitude of PA and vLTF. In contrast, markers of apnoea severity, including the hypoxic burden, did not add to the ability to predict the magnitude of PA or vLTF.

## Abstract (highlights)

- **What is the central question of this study?**
  The goal of this meta‐analysis was to provide insight into the mechanisms that impact the magnitude of progressive augmentation (PA) and ventilatory long‐term facilitation (vLTF) in order to predict the response to treatment with mild intermittent hypoxia in patients living with obstructive sleep apnoea.
- **What is the main finding and its importance**
  The findings suggest that peripheral chemoreflex sensitivity to hypoxia influences the magnitude of PA and vLTF. In addition, besides intermittent hypoxia, stimuli that activate neural pathways involved in both the arousal response and the initiation of respiratory plasticity might also contribute to enhancing the magnitude of PA or vLTF.

## 1 INTRODUCTION

Exposure to mild intermittent hypoxia during wakefulness elicits multiple forms of respiratory plasticity in humans, including progressive augmentation (PA) of the hypoxic ventilatory response and ventilatory long‐term facilitation (vLTF) (Mateika & Narwani, 2009; Puri, Panza et al., 2021). Progressive augmentation is typified by a gradual increase in the ventilatory response to hypoxia from the initial to final episode of an intermittent hypoxia protocol (Mateika & Narwani, 2009; Puri, Panza et al., 2021). Likewise, vLTF is characterised by a prolonged sustained increase in minute ventilation following exposure to brief intermittent periods of hypoxia (Mateika & Narwani, 2009; Puri, Panza et al., 2021). Both forms of plasticity have been observed frequently during wakefulness in healthy humans (Harris, Balasubramaniam et al., 2006; Wadhwa, Gradinaru et al., 2008; Lee, Badr et al., 2009; Syed, Lin et al., 2013), humans with obstructive sleep apnoea (Lee, Badr et al., 2009; Gerst, Yokhana et al., 2011; Yokhana, Gerst et al., 2012; Syed, Lin et al., 2013; Panza, Puri et al., 2022; Panza, Puri et al., 2022) and in individuals with spinal cord injury (Tester, Fuller et al., 2014).

The initiation of these forms of plasticity and their interaction likely has a role in modulating breathing instability associated with spinal cord injury and/or sleep apnoea (Mateika & Komnenov, 2017). If so, identifying variables that impact the magnitude of respiratory plasticity in response to treatment with mild intermittent hypoxia is important. A previous meta‐analysis showed that the hypoxic ventilatory response (a non‐invasive indicator of peripheral chemoreflex sensitivity) measured during wakefulness was the sole predictor of PA during exposure to intermittent hypoxia (Panza, Kissane et al., 2023). In addition, the hypoxic ventilatory response, in combination with the administered hypoxic burden (the hypoxic burden is based on the number and duration of hypoxic episodes and the intensity of hypoxia), predicted the magnitude of vLTF (Panza, Kissane et al., 2023).

Previous findings have also shown that the magnitude of PA and vLTF is greater in some individuals with obstructive sleep apnoea (OSA) compared to healthy controls matched for age and body mass index (Lee, Badr et al., 2009). Based on the findings outlined in the previous paragraph, it is possible that the magnitude of PA and vLTF in OSA participants is linked to the hypoxic ventilatory response. Modifications in the hypoxic ventilatory response may be of genetic origin (Weil, 2003) or the consequence of neural plasticity initiated by nightly exposure to intermittent hypoxia (Narkiewicz, Kato et al., 1999; Tun, Hida et al., 2000; Mateika & Narwani, 2009; Puri, Panza et al., 2021). Nightly exposure to intermittent hypoxia might enhance or blunt the hypoxic ventilatory response depending on the magnitude of the nightly hypoxic burden and the number of years of exposure to the burden (Mateika & Narwani, 2009). Indeed, studies completed in animals and humans indicate that repeated exposure to intermittent hypoxia is coupled to an increase in the magnitude of long‐term facilitation (LTF) (Gerst, Yokhana et al., 2011; MacFarlane, Vinit et al., 2018; Perim, Sunshine et al., 2021), which could be initiated by an increase in the hypoxic ventilatory response.

On the other hand, there are other mechanisms (e.g., loop gain and arousal threshold) (Panza, Alex et al., 2019) and hallmarks (intermittent arousal) linked to sleep apnoea that might predict the magnitude of PA and/or vLTF either independently of or in accordance with the hypoxic ventilatory response (see Discussion for further details). Thus, in the present investigation, we explored whether modifications in mechanisms (i.e., chemoreflex sensitivity and the arousal threshold) that cause OSA, coupled to parameters that characterise the severity of OSA, predict the magnitude of PA and vLTF in OSA patients during wakefulness. The parameters used to characterise the severity of sleep apnoea included both traditional indicators (i.e., apnoea/hypopnoea index) and recently ascertained novel indicators (i.e., hypoxic burden and arousal burden) of sleep apnoea severity (Azarbarzin, Sands et al., 2019; Azarbarzin, Labarca et al., 2024). The goal of this meta‐analysis was to provide physiological insight into the mechanisms that impact the magnitude of PA and vLTF, which ultimately could be used to predict the response (i.e., magnitude of PA and vLTF) to treatment with mild intermittent hypoxia.

## 2 METHODS

### 2.1 Participants

The data used in this study were obtained from 91 participants living with OSA who were enrolled in experiments completed in our laboratory from 2009 to 2025 (Lee, Badr et al., 2009; Gerst, Yokhana et al., 2011; Syed, Lin et al., 2013; Panza, Puri et al., 2022; Panza, Puri et al., 2022) and are available upon request. The Institutional Review Board of Wayne State University School of Medicine and John D. Dingell Veterans Affairs Medical Center approved the protocols (nos 010109M1FV, 070202M1F and 060112M1FV). The protocols conformed to the standards set by the *Declaration of Helsinki*. Three studies were registered at clinicaltrials.gov (NCT00860743, NCT05558501, NCT03736382). All participants provided written consent prior to enrolling in the study. The majority of participants were not treated with medication (*n* = 87), and the remaining participants were treated with a single blood pressure medication (*n* = 4). Moreover, participants were not treated with continuous positive airway pressure prior to completion of the baseline sleep study. All participants had normal lung function (forced vital capacity >80% of predicted values; FEV1.0/FVC > 70% of predicted values), a normal sleep–wake cycle (i.e., no shift work), and a sleep efficiency greater than 75%. The majority of participants did not have any associated co‐morbidities (*n* = 66), and the remaining participants (*n* = 25) were living with hypertension (blood pressure >130/80 mmHg).

### 2.2 Experimental protocols

#### 2.2.1 Sleep studies

In‐lab baseline sleep studies were completed, whilst participants slept in the supine position, to confirm the presence of sleep apnoea. Sleep was monitored with an electroencephalogram (C3/A2, C4/A1, O1/A2 and O2/A1), electrooculograms, submental electromyogram and an electrocardiogram. Abdominal and thoracic wall movements were measured with inductive plethysmography (Respitrace; Ambulatory Monitoring, Ardsley, NY, USA). Airflow, breathing frequency and inspiratory and expiratory volumes were obtained with a pneumotachometer (model RSS100‐HR; Hans Rudolph, Shawnee, KS, USA) connected to a nasal/facemask. End‐tidal oxygen (model 17515; Vacumed, Ventura, CA, USA) and end‐tidal carbon dioxide (model 17518; Vacumed) were obtained from expired air through sampling tubes connected to built‐in ports on the nasal/facemask. Mask pressure was measured by a port on the nasal mask, allowing the connection of a pressure transducer. In addition, epiglottic pressure was measured by using a transducer‐tipped catheter (Mikro‐Cath 825‐0101; Millar, Houston, TX, USA) to determine the nature of events, and the oxygen saturation was measured with a pulse oximeter (Biox 3700; Ohmeda, Boulder, CO, USA). All physiological parameters were converted from analog to digital at a sampling frequency of 100 Hz/channel and then fed into a computer using a commercially available software package (Gamma version 4.0; Astro‐Med, West Warwick, RI, USA).

#### 2.2.2 Intermittent hypoxia protocols

Following completion of the in‐lab baseline sleep study, participants completed one of two intermittent hypoxia protocols during wakefulness at the same time of day (i.e., morning). The protocols were similar in that a 10–15 min baseline period (B_1_) was initially established with participants breathing room air (Lee, Badr et al., 2009; Gerst, Yokhana et al., 2011; Syed, Lin et al., 2013; Panza, Puri et al., 2022; Panza, Puri et al., 2022). Thereafter, carbon dioxide was increased and a second baseline (B_2_) was established for 10–15 min (Lee, Badr et al., 2009; Gerst, Yokhana et al., 2011; Syed, Lin et al., 2013; Panza, Puri et al., 2022; Panza, Puri et al., 2022). The level that carbon dioxide was increased above baseline was 2–3 (2.74 ± 0.15) mmHg (Syed, Lin et al., 2013; Panza, Puri et al., 2022; Panza, Puri et al., 2022) or 3–4 (3.19 ± 0.16) mmHg (Lee, Badr et al., 2009; Gerst, Yokhana et al., 2011) on average. The established level of carbon dioxide was maintained throughout the remainder of the protocol. Both protocols included 12 episodes of hypoxia (Lee, Badr et al., 2009; Gerst, Yokhana et al., 2011; Syed, Lin et al., 2013; Panza, Puri et al., 2022) and the level of hypoxia achieved during each episode was 50 mmHg. The episodes were either 2 min (Syed, Lin et al., 2013; Panza, Puri et al., 2022; Panza, Puri et al., 2022) or 4 min (Lee, Badr et al., 2009; Gerst, Yokhana et al., 2011) in duration. The episodes were interspersed with recovery periods that were similar in length to the episode duration. The last hypoxic episode was followed by a final recovery period that was 30 min in duration (Lee, Badr et al., 2009; Gerst, Yokhana et al., 2011; Syed, Lin et al., 2013; Panza, Puri et al., 2022; Panza, Puri et al., 2022). Thus, Protocol 1 (P1) was characterised by 12–4 minute episodes of hypoxia with a carbon dioxide level sustained 3.19 ± 0.16 mmHg above baseline on average, whilst Protocol 2 (P2) was characterised by 12–2 Variable minute episodes of hypoxia with a carbon dioxide level sustained 2.74 ± 0.15 mmHg above baseline on average.

During the intermittent hypoxia protocols, minute ventilation, breathing frequency and tidal volume were collected on a breath‐by‐breath basis using commercially available software (LabVIEW, National Instruments, Austin, TX, USA). Subjects breathed through a tight‐fitting face mask throughout the entire protocol. The face mask was connected to a two‐way non‐rebreathing valve, which was connected to a 5‐way stop‐cock. Non‐diffusible bags with 8% oxygen were connected to the 5‐way stop‐cock. During hypoxic episodes, the valve was turned into the hypoxic mixture, and during the recovery periods, the valve was turned to room air. 100% oxygen and carbon dioxide were titrated into the inspirate to control end‐tidal gases according to the specific protocol. Respiratory variables were measured with a pneumotachograph in all protocols. Similarly, heart rate and oxygen saturation were monitored using a pulse oximeter, along with an electrocardiogram. The data was collected using commercially available software (LabVIEW, National Instruments, Austin, TX, USA; WinDaq, Dataq Instruments, Akron, OH, USA) at a sampling rate of 250 Hz.

### 2.3 Data analysis

#### 2.3.1 Sleep studies

All polysomnography studies were analysed for sleep stage, arousals and respiratory‐related events according to standard published criteria. An absence or a ≥90% reduction in airflow for a duration of ≥10 s was identified as an apnoea. A hypopnoea was defined by a ≥30% reduction in airflow that lasted for a minimum of 10 s. To be classified as a respiratory‐related event, the reduction in airflow was accompanied by either an arousal or a ≥3% drop in oxygen saturation in the absence of an arousal. Obstructive events were scored based on a progressive increase in ventilatory effort that was evident in epiglottic pressure measurements and in abdominal and thoracic wall movement. Arousals were identified by a significant increase in electroencephalography frequency and amplitude for ≥3 s. The start and end of arousals (i.e., arousal duration) were also determined.

Following identification of respiratory events and arousals, indices which included the apnoea/hypopnoea and arousal (respiratory and spontaneous arousals) index were calculated for non‐rapid eye movement sleep. To calculate the indices, the total number of identified events or arousals was divided by non‐rapid eye movement sleep time and expressed as the number of events per hour. In addition, a MATLAB (MathWorks, Natick, MA, USA) program that has been previously described was used to determine the hypoxic and arousal burden during sleep (Labarca, Vena et al., 2023; Martinez‐Garcia, Sánchez‐de‐la‐Torre et al., 2023). The hypoxic burden was calculated by summing the area under the respiratory event‐related oxygen desaturation curve associated with each detected breathing event, divided by total sleep time to determine the total amount of respiratory event‐related hypoxaemia over the sleep period (Azarbarzin, Sands et al., 2019). The arousal burden was defined as the cumulative duration of all arousal events expressed in minutes relative to total sleep time (Azarbarzin, Labarca et al., 2024). If an arousal terminated in a wake epoch, the arousal duration was calculated as the time between the arousal onset and the end of the related sleep epoch.

A MATLAB program, which has been previously described (Terrill, Edwards et al., 2015; Joosten, Leong et al., 2017; Landry, Andara et al., 2018), was also used to quantify loop gain, the arousal threshold and other parameters (see below) from the physiological measures obtained during the sleep studies. Briefly, the program was developed on the premise that obstructive breathing events provide a disturbance to the ventilatory control system, leading to increases in carbon dioxide and decreases in oxygen that augment the ventilatory drive (Terrill, Edwards et al., 2015). The increased ventilatory drive is reflected in the extent of hyperventilation that occurs after the airway is open following termination of an obstructive event. Accordingly, ventilatory drive is modelled as the sum of the ventilatory response to a chemical drive (i.e., changes in the partial pressure of oxygen and carbon dioxide) and a non‐chemical drive (i.e., wakefulness drive) that accompanies arousal (Terrill, Edwards et al., 2015). The time sequence of the ventilatory response to chemical stimuli is modelled with parameters that reflect the circulation time between the lung and chemoreceptors (i.e., time delay), the time course of carbon dioxide buffering in the lung and tissues (i.e., time constant), and the overall gain of the response (Terrill, Edwards et al., 2015). These temporal parameters, coupled with the ventilatory response to arousal, are modified until the modelled ventilatory drive closely fits the ventilation measured when the airway is not obstructed. These parameters are then used to compute the magnitude of loop gain. The ventilatory drive measured immediately before each arousal at the termination of a respiratory event was identified. The mean of the ventilatory drive values is considered to be the arousal threshold.

#### 2.3.2 Mild intermittent hypoxia during wakefulness

The hypoxic burden associated with each protocol administered during wakefulness was calculated by determining the area (base × height) of the decrease in the partial pressure of end‐tidal oxygen that was associated with each episode. Subsequently, the cumulative hypoxic burden was determined by summing the area calculated for each episode. Respiratory variables, end‐tidal gases, heart rate and oxygen saturation were averaged for the final 5 min of B_1_ and B_2_ for each protocol. For P1, the last 120 s of each hypoxic episode and subsequent recovery period were averaged. The final 30‐min end‐recovery period was divided into six 5‐min segments, and each variable was averaged for each segment. However, no difference existed between the segments; thus, the data collected for the entire 30‐min recovery were ultimately averaged to complete the statistical analysis. A similar analysis was completed for P2 with the exception that data collected over the last 30 s of each hypoxic episode and recovery period were averaged.

For each participant, the hypoxic ventilatory response was determined for the initial three and final three episodes of each protocol. The hypoxic ventilatory response was calculated by subtracting the ventilation recorded during the hypoxic episode from B_2_. The difference in ventilation was divided by the difference in the partial pressure of end‐tidal oxygen measured during B_2_ and each hypoxic episode. The responses measured during the first three and last three episodes were averaged. Thereafter, a regression line was fit to the average of the initial three and final three episodes for each participant, and the slope of the line was considered to be an indicator of the degree of PA. The hypercapnic ventilatory response was also determined for each participant. To obtain this measure, ventilation recorded during B_2_ was subtracted from B_1_, and this value was divided by the difference in the partial pressure of end‐tidal carbon dioxide between B_2_ and B_1_. The magnitude of ventilatory long‐term facilitation was determined by dividing the average ventilation measured over the 30‐min end‐recovery period by the ventilation measured during B_2_. Prior to standardising to baseline, the magnitude of ventilation during the end recovery period was corrected for any potential drift in carbon dioxide that might have occurred from baseline to end‐recovery for a given participant. For each participant, the magnitude of ventilation linked to a drift in carbon dioxide was calculated based on the hypercapnic ventilatory response that was determined for each individual. This correction was made to ensure that the magnitude of ventilatory long‐term facilitation was not overestimated because of a potential drift in carbon dioxide from baseline to the end of recovery. Ultimately, the magnitude of vLTF uncorrected and corrected was similar (1.34 ± 0.21 vs. 1.33 ± 0.22 standardised to baseline, *P* = 0.895).

### 2.4 Statistical analysis

Initially, a best subset regression analysis was completed to identify potential models that predict the magnitude of progressive augmentation and ventilatory long‐term facilitation. The variables included in the best subset regression analysis are listed in Table 1. Note that the hypoxic burden measured during sleep was log transformed (Azarbarzin, Sands et al., 2019). Two iterations of the analysis were completed. One version was completed using recently formulated novel indices of apnoea severity (i.e., hypoxic and arousal burden), whilst the other version was completed using standard indices of sleep apnoea severity. The two versions were completed to determine if similar predictive outcomes were evident, independent of the use of novel or standard indices of sleep apnoea severity. Likewise, completion of the two versions served to avoid multicollinearity between measures of the hypoxic and arousal burden and standard indices of apnoea severity. The model selected from the best subset regression analysis was based on three criteria: the highest adjusted *R* ^2^, the lowest mean square error, and the lowest Mallows’ *C*_p_ statistic. Following identification of the best subset model, a multiple linear regression analysis and a backward stepwise regression model were used to identify the most impactful variables in the model, thereby simplifying the model whilst retaining its predictive accuracy. The magnitude of PA or vLTF was considered the dependent variable, and the variables identified using the best subset regression analysis were included as independent variables in the multiple linear and stepwise regression model. Significance was set at *P* < 0.05, and the data were presented as means ± standard deviation.

**TABLE 1:** Anthropometric and physiological measures.

Variable	Value
*n*	91
Age (years)^*^, ^†^	31.7 ± 9.9
Body mass index (kg/m^2^)^*^, ^†^	28.3 ± 4.2
Race^*^, ^†^	45 W, 58 AA, 6 A, 3 H
Sex	82 males, 9 females
Apnoea/hypopnoea index (events/h)^*^	46.0 ± 26.1
Apnoea index (events/h)	17.7 ± 21.1
Hypopnoea index (events/h)	28.4 ± 17.3
Lowest $S_{aO_{2}}$ during events (%)	86.2 ± 6.2
Mean $S_{aO_{2}}$ during events (%)^*^	92.5 ± 2.1
Respiratory arousal index	39.1 ± 23.4
Total arousal index (arousals/h)^*^	60.7 ± 23.6
Apnea duration (s)^*^	18.6 ± 5.0
Hypopnoea duration (s)	20.7 ± 4.6
Loop Gain_1.0_ ^*^, ^†^	0.53 ± 0.14
Ventilatory response to arousal^*^, ^†^	0.18 ± 0.16
Arousal threshold^*^, ^†^	1.2 ± 0.24
Hypoxic burden (sleep – % minute/h)^†^	45.5 ± 58.1
Arousal burden (% total sleep time)^†^	11.9 ± 5.8
Hypoxic burden (wake – minute × mmHg)^*^, ^†^	2406.6 ± 70.2 (P1) 1234.7 ± 91.7 (P2)
HVR (wake) (L/min/mmHg)^*^, ^†^	0.17 ± 0.08
Progressive augmentation (wake)	0.046 ± 0.055
LTF corrected (wake) (standardised to baseline)	1.33 ± 0.23

* Variables, including established measures of apnoea severity, which were used to complete the initial version of the best subset regression analysis.

^†^ Variables, including more recently established markers of apnoea severity, used to complete a second version of the analysis. A, Asian; AA, African‐American; H, Hispanic; W, White.

## 3 RESULTS

**FIGURE 1:**
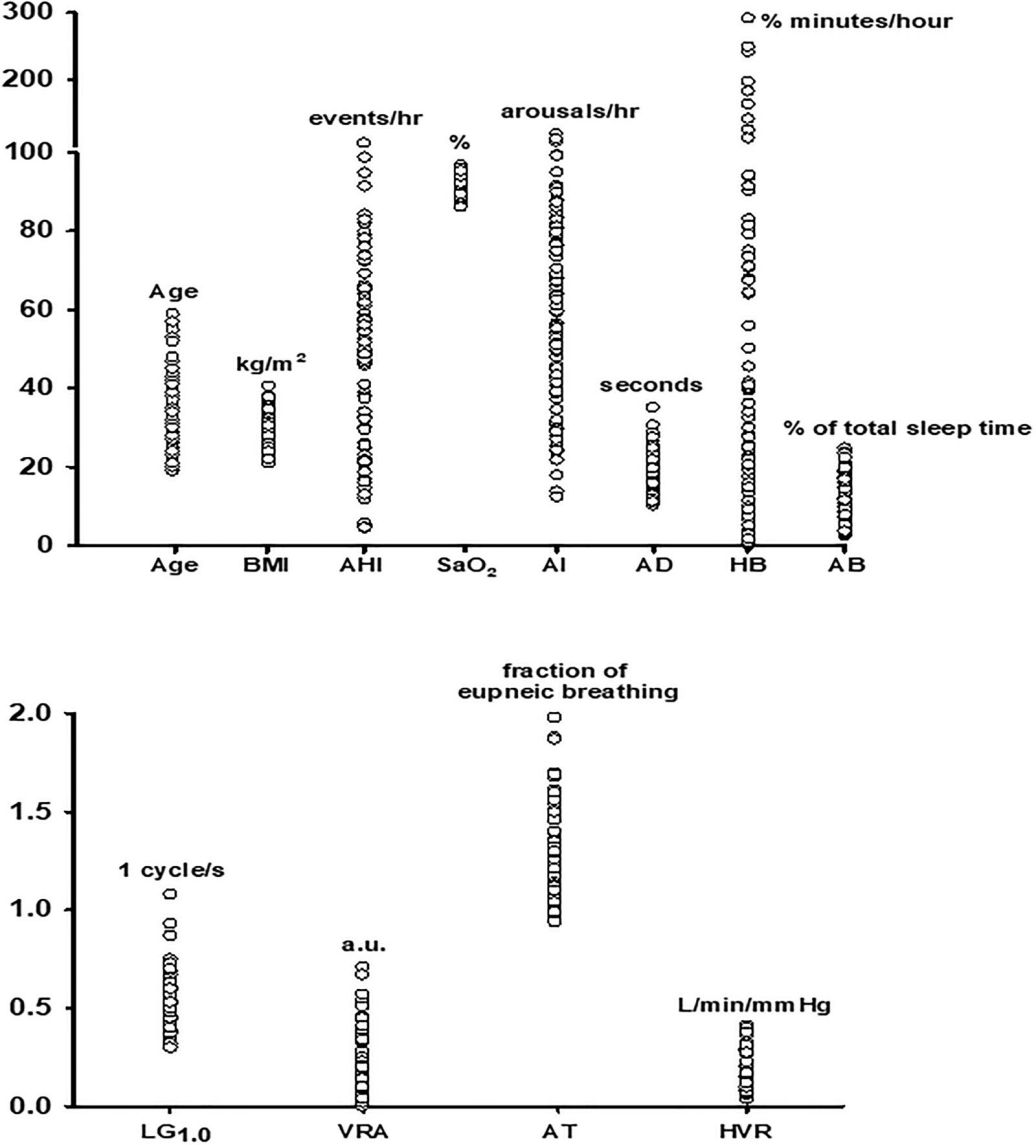
Scatterplots showing individual data points for each participant. The variables shown were used in the best subset regression analyses. AB, arousal burden; AD, apnoea duration; AHI, apnoea/hypopnoea index; AI, total arousal index; AT, arousal threshold; BMI, body mass index; HB, hypoxic burden; HVR, hypoxic ventilatory response; LG_1.0_, loop gain; $S_{aO_{2}}$, mean oxygen saturation during an event; and VRA, ventilatory response to arousal.

Figure 1 shows the anthropometric variables, endotypic markers (i.e., loop gain and arousal threshold) and indicators of OSA severity that were measured from each participant exposed to mild intermittent hypoxia. Table 1 shows the mean ± standard deviation calculated using the individual data shown in Figure 1.

**FIGURE 2:**
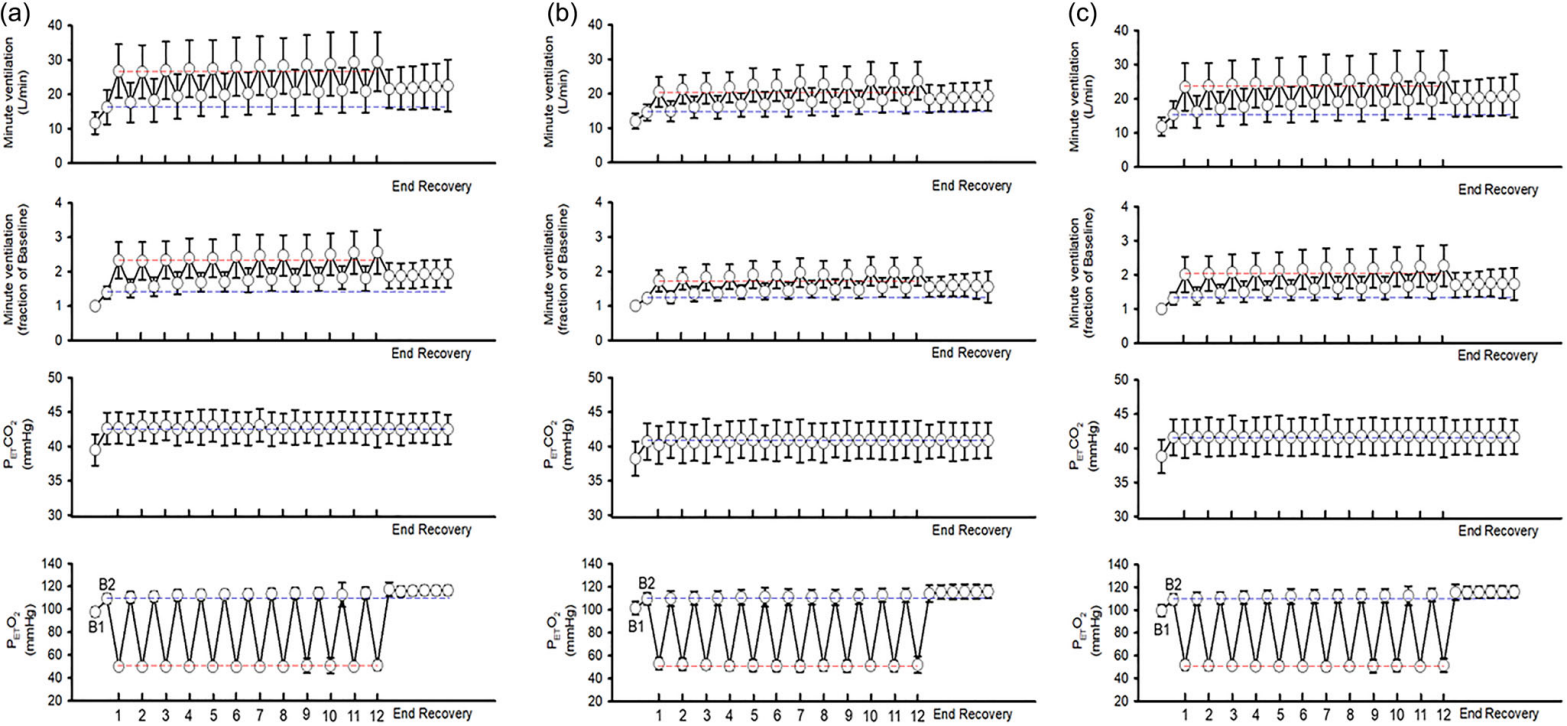
Mean values of absolute and standardised minute ventilation, partial pressure of end‐tidal carbon dioxide ($P_{\mathrm{ETC}O_{2}}$) and partial pressure of end‐tidal oxygen ($P_{\mathrm{ET}O_{2}}$) recorded during Protocol 1 (P1; a), Protocol 2 (P2; b) and a composite of P1 and P2 (c). The data shown include (i) the last 5 min of baseline 1 (B_1_) and baseline 2 (B_2_), (ii) the last 2 min of each episode of hypoxia in P1 and the last 30 s of each episode in P2 (indicated by vertical line on x‐axis), (iii) the last 2 min (P1) or 30 s (P2) of each normoxic period that separated the hypoxic episodes, and (iv) each 5 min segment of the end‐recovery period. Note in (a, b, c) that progressive augmentation and vLTF is evident. Specifically, minute ventilation during the end‐recovery period is above the dashed blue line, which demarcates baseline measures. In addition, the ventilatory response to hypoxia during the last episode is above the red line, which demarcates the ventilatory response to hypoxia during the first episode.

Figure 2a shows mean absolute values of minute ventilation and mean standardised (expressed as a fraction of B_2_) values of minute ventilation, along with the partial pressure of end‐tidal carbon dioxide and oxygen during B_1_, B_2_, each hypoxic episode and each recovery period of P1. Figure 2b shows the identical variables recorded during the completion of P2, whilst Figure 2c is a composite of the data collected during P1 and P2. Figure 2c shows that during the protocol, the partial pressure of end‐tidal carbon dioxide between B_2_ and the end‐recovery period was strictly controlled, as was the targeted hypoxic intensity.

**FIGURE 3:**
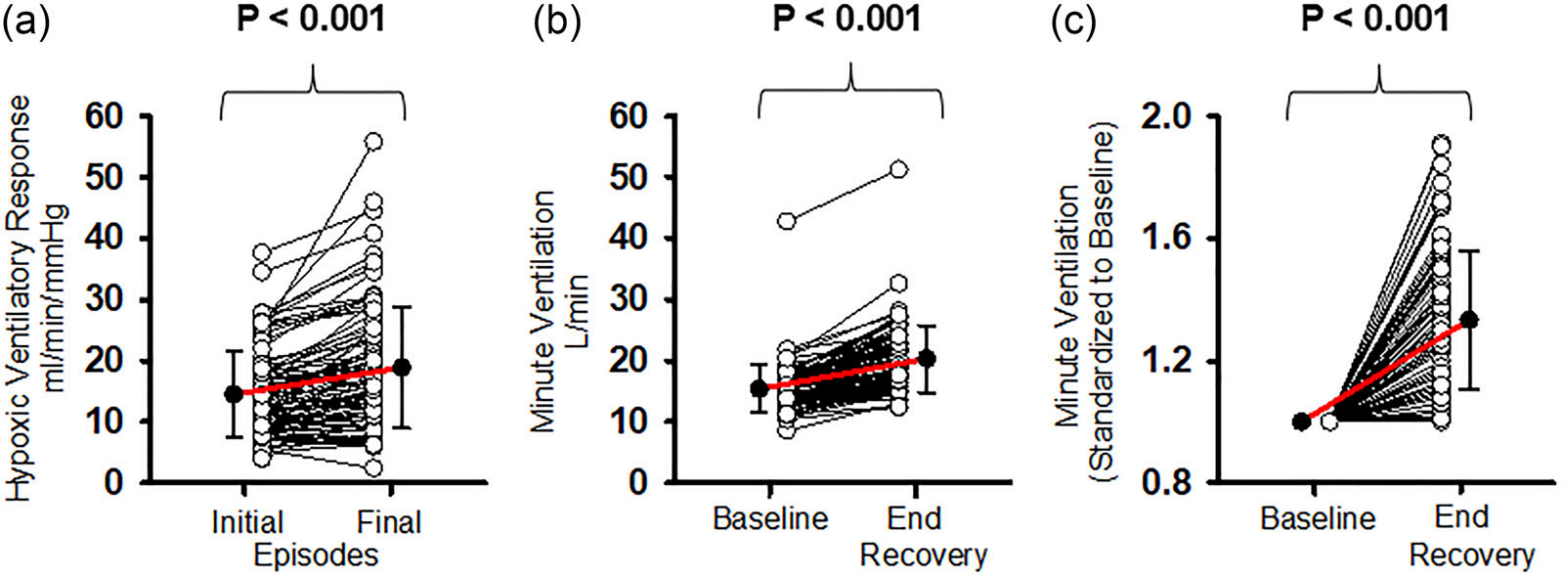
Scatterplots showing individual data points for each participant. (a) Mean values of the hypoxic ventilatory response measured from the initial and final 3 episodes of the intermittent hypoxia protocols. Note that the slope of the line connecting measures of the hypoxic ventilatory response recorded during the initial episodes and final episodes (see red line) is indicative of the magnitude of progressive augmentation. (b) Mean values of absolute minute ventilation measured during baseline (B_2_) and the end recovery period. (c) Mean values of minute ventilation during the end recovery period that were standardised to baseline values. Student's paired *t*‐test was used to compare the data measured during the initial and final episodes or baseline and end‐recovery periods (*n* = 91).

Figure 3 shows the individual data points associated with the mean values shown in Figure 2c. Specifically, Figure 3a shows for each participant the mean hypoxic ventilatory response measured during the initial three and final three episodes of the protocol. In addition, Figures 3b and 3c shows for each participant the minute ventilation measured during B_2_ and the end recovery period shown in Figure 2c. Absolute values of minute ventilation are shown in Figure 3b, whilst measures of minute ventilation standardised to baseline (fraction of baseline) are shown in Figure 3c.

The variables identified by an asterisk and dagger shown in Table 1 were used to complete a best subset regression analysis to determine the variables that best predicted the magnitude of PA or vLTF. The variables identified by an asterisk are established measures of apnoea severity, which were used to complete the initial version of the best subset regression analysis. More recently established markers of apnoea severity, identified by the dagger, were used to complete a second version of the analysis. The variables identified by both an asterisk and a dagger were used in both analyses.

Supporting information Table S1 shows the matrix of the best subset regression analysis used to predict the magnitude of PA using novel indicators of apnoea severity (i.e., hypoxic burden and arousal burden). The results show that the best subset included the hypoxic ventilatory response, hypoxic burden (sleep), arousal threshold and body mass index (Table S1, Model 4). The multi‐linear regression analysis that included the identified subset of variables showed that the magnitude of PA was predicted solely by the hypoxic ventilatory response (Table 2, top). The inclusion of the hypoxic burden measured during sleep, the arousal threshold and body mass index did not significantly add to the ability of the equation to predict the magnitude of PA. The results of the backward stepwise regression analysis revealed that the hypoxic ventilatory response (*P* < 0.001) and body mass index (*P* = 0.033) both contributed to predicting the magnitude of progressive augmentation.

**TABLE 2:** Predictors of the magnitude of progressive augmentation and long‐term facilitation using novel indicators of hypoxic and arousal burden.

	*R*	Coefficient	SE	*t*	*P*	VIF
Progressive augmentation (*n* = 91)	0.589					
Constant		−0.0567	0.0417	−1.362	0.177	
Hypoxic ventilatory response		0.381	0.0613	6.207	< 0.001	1.051
Hypoxic burden (sleep)		0.00913	0.00762	1.199	0.234	1.292
Arousal threshold		−0.0324	0.0220	−1.469	0.146	1.174
Body mass index		0.00237	0.00128	1.854	0.067	1.187
LTF magnitude (*n* = 91)	0.602					
Constant		1.040	0.102	10.150	<0.001	
Hypoxic ventilatory response		1.028	0.303	3.390	0.001	1.551
Hypoxic burden (wake)		0.00008	0.000045	1.770	0.080	1.760
Arousal burden		0.018	0.005	3.372	0.001	2.283
Hypoxic burden (sleep)		−0.0645	0.038	−1.696	0.094	1.943
Loop gain		−0.276	0.162	−1.700	0.093	1.225

Supporting information Table S2 shows the matrix of the best subset regression analysis used to predict the magnitude of vLTF using novel indicators of apnoea severity. Based on set criteria (see ‘Statistical analysis’ for the criteria used), the regression analysis indicated that a subset which included the hypoxic ventilatory response, hypoxic burden (wake), arousal burden, hypoxic burden (sleep) and loop gain (Table S2, Model 5) best predicted the magnitude of vLTF. The multi‐linear regression analysis that included the identified subset of variables showed that the magnitude of vLTF was predicted by a linear combination of the hypoxic ventilatory response and the arousal burden (Table 2, bottom). The inclusion of hypoxic burden (wake and sleep) and loop gain did not significantly add to the ability of the equation to predict the magnitude of vLTF (Table 2, bottom). This result was supported by the backward stepwise regression analysis, which revealed identical findings (hypoxic ventilatory response, *P* < 0.001; arousal burden, *P* < 0.015).

Supporting information Table S3 shows the results of the best subset regression model used to predict the magnitude of PA using established measures of apnoea severity. The results show that the best subset included the hypoxic ventilatory response, ventilatory response to arousal, apnoea duration, arousal index, body mass index and age (Table S3, Model 6). The multi‐linear regression analysis that included the identified subset of variables showed that the magnitude of PA was predicted by a combination of the hypoxic ventilatory response and arousal index (Table 3, top). The inclusion of the ventilatory response to arousal, apnoea duration, body mass index and age did not significantly add to the ability of the equation to predict the magnitude of PA. This result was supported by the backward stepwise regression analysis, which revealed identical findings (hypoxic ventilatory response, *P* < 0.001; arousal index, *P* < 0.021).

**TABLE 3:** Predictors of the magnitude of progressive augmentation and long‐term facilitation using standard indicators of sleep apnoea.

	*R*	Coefficient	SE	*t*	*P*	VIF
Progressive augmentation (*n* = 91)	0.625					
Constant		−0.0443	0.0411	−1.076	0.285	
Hypoxic ventilatory response		0.330	0.0660	5.001	<0.001	1.212
Ventilatory response to arousal		−0.0620	0.0339	−1.831	0.071	1.217
Apnoea duration		−0.00160	0.00111	−1.433	0.156	1.074
Arousal index		0.000695	0.000281	2.475	0.015	1.753
Body mass index		0.002207	0.00144	1.440	0.154	1.512
Age		−0.000776	0.000669	−1.159	0.250	1.810
LTF magnitude (*n* = 91)	0.593					
Constant		1.045	0.104	10.019	<0.001	
Hypoxic ventilatory response		1.159	0.295	3.928	<0.001	1.446
Hypoxic burden (wake)		0.00007	0.00005	1.522	0.132	1.841
Ventilatory response to arousal		−0.178	0.149	−1.195	0.236	1.411
Loop gain		−0.385	0.164	−2.346	0.021	1.234
Arousal index		0.0034	0.0011	3.191	0.002	1.560

Supporting information Table S4 shows the results of the best subset regression model used to predict the magnitude of vLTF using established measures of apnoea severity. The results show that the best subset included the hypoxic ventilatory response, hypoxic burden (wakefulness), ventilatory response to arousal, loop gain and the arousal index (Table S4, Model 5). The multi‐linear regression analysis that included the identified subset of variables showed that the magnitude of vLTF was predicted by a combination of the hypoxic ventilatory response, loop gain and the arousal index (Table 3, bottom). The inclusion of the hypoxic burden associated with the protocol and the ventilatory response to arousal did not significantly add to the ability of the equation to predict the magnitude of vLTF (Table 3, bottom). This result was supported by the backward stepwise regression analysis, which revealed identical findings (hypoxic ventilatory response, *P* < 0.001; loop gain, *P* < 0.038; arousal index, *P* < 0.007).

## 4 DISCUSSION

The present investigation was designed to identify anthropometric measures, mild intermittent hypoxia protocol elements and indicators of apnoea severity that predict the magnitude of PA and vLTF in participants with OSA. Our results showed that the hypoxic ventilatory response was a strong predictor of the magnitude of PA and vLTF. In addition, our findings showed that novel and standard indices of arousal (i.e., arousal burden and arousal index) in patients with sleep apnoea also contributed to predicting the magnitude of PA and vLTF. In contrast, novel (hypoxic burden) and standard (i.e., apnoea hypopnoea index, apnoea duration, oxygen saturation during apnoea) indicators of apnoea severity did not add to the ability to predict the magnitude of PA and vLTF. To ensure that the selected models were robust and to guard against potential overfitting, we carefully chose the most relevant predictor variables that met specific criteria (see ‘Statistical analysis’ for criteria) following completion of the best subset regression analysis. In addition, we ensured that we had a sufficient number of observations (i.e., *n* = 91) that were equivalent to at least 15 observations for each model predictor. Lastly, the calculation of predicted *R* ^2^ was used as a cross‐validation method. In regard to the cross‐validation method, we followed the common guideline that the difference in the predicted *R* ^2^ and adjusted *R* ^2^ was less than 0.2.

### 4.1 Predicting the magnitude of PA and vLTF

We previously completed a meta‐analysis that was designed to explore the impact of anthropometric measures and mild intermittent hypoxia protocol elements on the magnitude of PA and vLTF (Panza, Kissane et al., 2023). The magnitude of PA and vLTF was measured from one of three protocols which varied in regard to episode number (8 vs. 12 episodes), episode duration (2 vs. 4 min) and the level of carbon dioxide maintained above baseline (i.e., the level of carbon dioxide maintained above baseline varied from 2 to 5 mmHg across protocols) (Panza, Kissane et al., 2023). On the other hand, the targeted hypoxic intensity achieved for each protocol (i.e., partial pressure of end‐tidal oxygen ∼50 mmHg) and the time of day the protocol was administered (i.e., early morning during wakefulness) were similar (Panza, Kissane et al., 2023). Our previous results showed that the magnitude of PA was predicted solely by the hypoxic ventilatory response. In other words, a greater hypoxic ventilatory response was correlated with an increased magnitude of PA. Our results also showed that the magnitude of vLTF was predicted by a combination of the hypoxic ventilatory response and the hypoxic burden associated with the mild intermittent hypoxia protocol (Panza, Kissane et al., 2023).

Nonetheless, we did not explore whether mechanisms and hallmarks of sleep apnoea impact the magnitude of PA and vLTF. Markers of sleep apnoea were not included as variables in the regression analysis because sleep studies were not completed in 25% of the participants, and we elected to maintain a complete data set for the analysis (Panza, Kissane et al., 2023). Moreover, although standard measures of apnoea severity (apnoea/hypopnoea index, apnoea duration, oxygen saturation) were available from the sleep studies that were completed, contemporary indicators of endotypic mechanisms (arousal threshold and loop gain) linked to sleep apnoea, along with new‐found indicators of apnoea severity (arousal burden and hypoxic burden) that might correlate with the magnitude of PA and/or vLTF were not available.

The potential impact that variables linked to apnoea severity might have on the magnitude of PA and vLTF is based on findings obtained from both animal and human studies. Studies completed in animals have shown that the magnitude of phrenic long‐term facilitation is enhanced following repeated daily exposure to intermittent hypoxia (MacFarlane, Vinit et al., 2018; Perim, Sunshine et al., 2021). Similarly, repeated daily exposure to mild intermittent hypoxia enhances the magnitude of PA and vLTF in humans with OSA (Gerst, Yokhana et al., 2011). Moreover, the magnitude of PA and/or vLTF is greater in participants with sleep apnoea compared to healthy control participants matched for age, sex and body mass index (Lee, Badr et al., 2009). Collectively, these findings lead to the hypothesis that nightly exposure to mild intermittent hypoxia might be responsible for the enhanced PA and vLTF that we previously observed in participants living with obstructive sleep apnoea compared to healthy controls (Lee, Badr et al., 2009). However, there was limited support to link repeated exposure to nocturnal episodes of hypoxia with the magnitude of PA and vLTF.

### 4.2 Hypoxic ventilatory response

The present investigation is an initial step exploring whether repeated nocturnal exposure to hallmarks of sleep apnoea (i.e., arousal and intermittent hypoxia) is linked to the magnitude of PA and vLTF in humans. Two iterations of a subset regression analysis, which included either novel or standard markers of apnoea severity, were employed to reveal an initial set of variables that predict the magnitude of PA or vLTF. One iteration included novel indicators of apnoea severity, and the other iteration included standard markers of apnoea severity. The outcome of the two iterations was similar in that the hypoxic ventilatory response (NB measured during wakefulness) was identified as a predictor of both PA and vLTF. The subset regression analysis also identified loop gain (NB measured during sleep) as a potential predictor of vLTF. It is not surprising that the hypoxic ventilatory response and loop gain were identified as potential predictors of the magnitude of vLTF, since controller gain (i.e., chemoreflex sensitivity) is a component of loop gain, and we previously showed that the hypoxic ventilatory response measured during wakefulness and loop gain measured during sleep are correlated (Puri, Panza et al., 2023).

Following identification of the best subset model, a multiple linear and backward stepwise regression model was used to identify the most impactful variables in the model, thereby simplifying the model whilst retaining its predictive accuracy. These analyses showed that the hypoxic ventilatory response remained as a predictor of the magnitude of PA and vLTF, which is similar to the result reported in our previous investigation (Panza, Kissane et al., 2023). From a mechanistic point of view, the hypoxic ventilatory response is thought to be a non‐invasive indicator of peripheral chemoreflex sensitivity, although oxygen sensors have been located elsewhere within the central nervous system more recently (Gourine & Funk, 2017; Barioni, Derakhshan et al., 2022). Previous work has shown that stimulation of the peripheral chemoreceptors has a role in the initiation of PA and/or LTF in animals (Fregosi & Mitchell, 1994; Cummings & Wilson, 2005; Fuller, 2005; Sokolowska & Pokorski, 2006) and humans (Vermeulen, Benbaruj et al., 2020). Moreover, exposure to mild intermittent hypoxia initiates LTF via feedback from the peripheral chemoreceptors to neuronal groups in the brainstem (Millhorn, Eldridge et al., 1980; Millhorn, 1986; Erickson & Millhorn, 1994; Morris, Arata et al., 1996; Morris, Arata et al., 1996; Mitchell, Baker et al., 2001; Huxtable, Macfarlane et al., 2014) (see ‘Arousal burden’ for discussion of neuronal groups). Thus, increased chemoreflex sensitivity to hypoxia might translate into a more potent stimulus that ultimately induces an increase in the magnitude of PA and vLTF. An increase in chemoreflex sensitivity may be of genetic origin (Weil, 2003) or the consequence of neural plasticity initiated by nightly exposure to intermittent hypoxia (Narkiewicz, Kato et al., 1999; Tun, Hida et al., 2000; Mateika & Narwani, 2009; Puri, Panza et al., 2021). Based on work completed in animal and human models, an increase in chemoreflex sensitivity might be expected in younger humans with sleep apnoea who are exposed to mild nocturnal intermittent hypoxia (infrequent short apnoeas coupled with mild levels of hypoxia). Conversely, blunting of chemoreflex sensitivity might be expected in older individuals with untreated sleep apnoea for many years who are exposed to severe hypoxic burdens (frequent long apnoeas coupled to severe levels of hypoxia). Additional studies are required to address these possibilities.

### 4.3 Hypoxic burden

The hypoxic burden associated with a given intermittent hypoxia protocol administered during wakefulness reflects the duration and intensity of the hypoxic stimulus activating the peripheral chemoreceptors, and theoretically the potency of feedback from this receptor to brainstem neuronal groups involved in the initiation of PA and/or vLTF. The results from our previous study showed that the protocol hypoxic burden contributed to predicting the magnitude of vLTF. Likewise, in the present investigation, one of the variables in the model generated by the subset regression analysis that predicted the magnitude of vLTF included the protocol hypoxic burden. However, following completion of the multiple linear and stepwise regression models, the hypoxic burden did not add to the ability of the equation to predict the magnitude of vLTF. The reason that the hypoxic burden played a smaller role in predicting the magnitude of vLTF in the present investigation is likely two‐fold. The two protocols that were employed in the present investigation were similar in regard to both the number of episodes and the hypoxic intensity, whilst episode duration differed. The similarities between the two protocols, coupled with the variables measured during sleep that were not included in the analysis completed in the previous investigation, likely led to the protocol hypoxic burden being less impactful than previously reported. Presumably, greater variations in the hypoxic burden across a number of protocols, administered over a varying number of days, would likely be more impactful than the protocol burdens included in the present study.

Despite this finding, we expected that the degree of nocturnal exposure to intermittent periods of hypoxia (e.g., hypoxic burden) during sleep would contribute to predicting the magnitude of PA and vLTF. This postulation was based on the range of hypoxic burdens measured across our sample of participants, coupled with the likelihood that our participants were exposed to the measured hypoxic burden nightly over many years. In line with this postulation, previous work has indicated that repeated exposure to mild intermittent hypoxia enhances the magnitude of phrenic long‐term facilitation in animals (MacFarlane, Vinit et al., 2018; Perim, Sunshine et al., 2021) or vLTF in humans (Gerst, Yokhana et al., 2011). Completion of the subset regression analysis provided initial support for our postulation, given that the hypoxic burden measured during sleep was included as a variable in the model that best predicted the magnitude of PA and vLTF. However, unexpectedly, the multiple linear and step‐wise regression analysis showed that the hypoxic burden measured during sleep, or other indices that might reflect nocturnal exposure to hypoxia (e.g., apnoea hypopnoea index, mean oxygen saturation during an event), was not required to accurately predict the magnitude of PA or vLTF.

### 4.4 Arousal burden

Instead of the hypoxic burden serving as a major predictor of the magnitude of PA and vLTF, our analyses indicated that a number of arousal indices might serve as predictors of the magnitude of PA and vLTF. More specifically, one iteration of the subset analysis indicated that the arousal threshold contributed to predicting PA. Likewise, the analysis, which incorporated standard makers of arousal (i.e., arousal index), indicated that the ventilatory response to arousal, along with the arousal index, contributed to predicting PA. Moreover, the arousal burden or arousal index was identified as a predictor of the magnitude of vLTF. Following completion of the multiple linear and stepwise regression analyses, it was clear that the most impactful arousal indices that predicted the magnitude of PA and vLTF were the arousal burden (i.e., vLTF) and arousal index (PA and vLTF).

We did not anticipate that indices of arousal severity (i.e., arousal burden or arousal index) would be a major predictor of the magnitude of vLTF. Nonetheless, our finding suggests that stimuli other than repeated daily exposure to mild intermittent hypoxia have a role in enhancing the magnitude of PA and/or vLTF. This suggestion aligns with mechanistic pathways known to be involved in both transient arousal from sleep and in initiating LTF in animals. Arousal from sleep involves a series of neuronal groups with ascending and descending projections within the central nervous system that are activated in response to a variety of stimuli, including changes in blood gases that accompany apnoeic events and non‐respiratory related stimuli (e.g., auditory and tactile) (Horner & Peever, 2017). Two of the neuronal groups involved in the arousal response are the locus coeruleus and dorsal raphe nucleus, which release noradrenaline and serotonin, respectively (Horner & Peever, 2017). Indeed, the absence of serotonin in the central nervous system results in a blunted arousal response during non‐rapid eye movement sleep (Solarewicz, Angoa‐Perez et al., 2015). The locus coeruleus and dorsal raphe nucleus have spinal projections to phrenic motoneurons. Moreover, studies have shown that both groups are involved in the initiation of LTF (Millhorn, Eldridge et al., 1980; Millhorn, 1986; Erickson & Millhorn, 1994; Morris, Arata et al., 1996; Morris, Arata et al., 1996; Mitchell, Baker et al., 2001; Huxtable, Macfarlane et al., 2014). Thus, these neuronal projections may mediate the impact of repeated daily nocturnal exposure to intermittent arousal on the magnitude of PA and vLTF.

We suggest that the arousal burden, rather than the hypoxic burden, is a more accurate predictor of the magnitude of PA or vLTF because it encapsulates all potential intermittent stimuli (e.g., respiratory, acoustic, tactile) that trigger at least two neuronal groups (i.e., locus coeruleus and dorsal raphe nucleus) known to be involved in both arousal and the initiation of LTF. In contrast, the hypoxic burden is a marker of a single stimulus that activates the same mechanistic pathway. Consequently, the hypoxic burden might not fully reflect the degree to which these pathways are intermittently activated throughout the night in individuals with sleep apnoea. Based on our postulation, arousal intensity and duration more accurately reflect the strength of the stimulus responsible for activating the neuronal pathways involved in enhancing the magnitude of vLTF, compared to the intensity of exposure and/or duration of exposure to hypoxia alone. In support of this contention, previous work has revealed that changes in blood gases associated with a given apnoeic event only account for a portion of the arousal response (Horner, Rivera et al., 2001). Consequently, other stimuli linked to apnoeic events must contribute to the magnitude of the arousal response (Horner, Rivera et al., 2001). Moreover, enhancement of the magnitude of PA and vLTF might also be dependent on whether repeated daily intermittent exposure to arousal stimuli occurs during sleep rather than wakefulness (Horner, Sanford et al., 1997). Studies have shown that the startle response during wakefulness is greater immediately following an arousal from sleep compared to the response recorded during wakefulness long after an arousal has occurred (Horner, Sanford et al., 1997). In other words, the impact of a given stimulus that leads to arousal and serves as input to the mechanistic pathways responsible for enhancing LTF is likely strongest when associated with the transition from sleep to wakefulness.

### 4.5 Summary and conclusion

In conclusion, the hypoxic ventilatory response and markers of arousal were identified as strong predictors of the magnitude of PA and vLTF in humans living with OSA. In contrast, markers of apnoea severity, including the hypoxic burden, did not significantly add to the ability to predict the magnitude of PA or vLTF. Our findings suggest that peripheral chemoreflex sensitivity to hypoxia might contribute to enhancing the magnitude of PA and vLTF. Likewise, repeated nocturnal exposure to stimuli that activate neural pathways involved in both the arousal response and the initiation of respiratory plasticity, besides intermittent hypoxia, might also contribute to enhancing the magnitude of PA or vLTF. These findings are an initial step towards identifying the physiological variables that impact the magnitude of PA and vLTF in humans living with OSA. Further studies are also necessary to explore whether increases in the magnitude of respiratory plasticity are also coupled with modifications of similar magnitude in other physiological responses (e.g., autonomic and cardiovascular responses).

## AUTHOR CONTRIBUTIONS

The data presented in this manuscript were collected from experiments completed in the laboratory of Jason H. Mateika. Conception of the work: Jason H. Mateika Contributed to the acquisition, analysis, or interpretation of data and drafting the work or revising it critically for important intellectual content: Jason H. Mateika, Danny Hammo, Dylan M. Kissane and Ali Azarbarzin. All authors have read and approved the final version of this manuscript and agree to be accountable for all aspects of the work in ensuring that questions related to the accuracy or integrity of any part of the work are appropriately investigated and resolved. All persons designated as authors qualify for authorship, and all those who qualify for authorship are listed.

## CONFLICT OF INTEREST

A.A. has received consulting fees from Respicardia, Eli Lilly, Amgen, Inspire, Cerebra and Apnimed. A.A. has a patent pending for phenotyping sleep apnoea using wearables (all unrelated to the submitted work). Apnimed is developing pharmacological treatments for Obstructive Sleep Apnea. A.A.’s interests were reviewed by Brigham and Women's Hospital and Mass General Brigham in accordance with their institutional policies. The other authors have declared no conflicts of interest.

## Supporting information



Tables S1–S4.

## Data Availability

The data used in this study were obtained from participants enrolled in experiments completed in our laboratory from 2009 to 2024. The inclusion of all data in a supporting information section could compromise ethical standards. However, the availability of data will be considered upon request.
