# Supplementary material for: The hypoxic ventilatory response and arousal burden predict the magnitude of ventilatory long‐term facilitation in humans with obstructive sleep apnoea
Source: Exp Physiol. 2025 Nov 6;111(3):1441–53. doi: 10.1113/EP093163 (PMC12949180; doi:10.1113/EP093163)
Supplement: Supplementary file 1 — Tables S1–S4. [file EPH-111-1441-s001.pdf]

SUPPLEMENTARY DATA

TABLE S1

A = Hypoxic Ventilatory Response
B = Hypoxic Burden (wake)
C = Arousal Burden
D = Hypoxic Burden (sleep)
E = Arousal Threshold
F = Ventilatory Response to Arousal
G = Loop Gain
H = Body mass index
I = Age

Model	Cp	Adjusted R^2	MSerr	A	B	C	D	E	F	G	H	I
1	2.892	0.277	0.002	*								
2	0.333	0.306	0.002	*							*	
3	1.046	0.309	0.002	*				*			*	
4	1.666	0.312	0.002	*			*	*			*	
5	2.965	0.310	0.002	*			*	*	*		*	
6	4.459	0.306	0.002	*			*	*	*		*	*
7	6.098	0.301	0.002	*		*	*	*	*		*	*
8	8.008	0.293	0.002	*		*	*	*	*	*	*	*
9	10.000	0.284	0.002	*	*	*	*	*	*	*	*	*

Cp – Mallow Cp statistic, MSerr – Model Selection Error Rate

TABLE S2

A = Hypoxic Ventilatory Response
B = Hypoxic Burden (wake)
C = Arousal Burden
D = Hypoxic Burden (sleep)
E = Arousal Threshold
F = Ventilatory Response to Arousal
G = Loop Gain
H = Body mass index
I = Age

Model	Cp	Adjusted R^2	MSerr	A	B	C	D	E	F	G	H	I
1	8.761	0.244	0.039	*								
2	4.535	0.286	0.037	*		*						
3	3.349	0.304	0.036	*		*				*		
4	3.728	0.309	0.036	*	*	*				*		
5	2.958	0.324	0.035	*	*	*	*			*		
6	4.377	0.321	0.035	*	*	*	*	*		*		
7	6.139	0.315	0.036	*	*	*	*	*	*	*		
8	8.092	0.306	0.036	*	*	*	*	*	*	*		*
9	10.000	0.299	0.036	*	*	*	*	*	*	*	*	*

Cp – Mallow Cp statistic, MSerr – Model Selection Error Rate

TABLE S3

A = Hypoxic Ventilatory Response
B = Hypoxic Burden (wake)
C = Arousal Threshold
D = Ventilatory Response to Arousal
E = Loop Gain
F = Apnea Duration
G = Arousal Index
H = Average SaO_2 during event
I = Apnea/Hypopnea Index
J = Body Mass Index
K = Age

Model	Cp	Adjusted R^2	MSerr	A	B	C	D	E	F	G	H	I	J	K
1	6.306	0.276	0.002	*										
2	2.760	0.313	0.002	*						*				
3	2.213	0.326	0.002	*			*			*				
4	1.630	0.340	0.002	*			*		*	*				
5	2.516	0.341	0.002	*			*		*	*			*	
6	3.237	0.344	0.002	*			*		*	*			*	*
7	4.952	0.338	0.002	*		*	*		*	*			*	*
8	6.664	0.332	0.002	*	*	*	*		*	*			*	*
9	8.300	0.327	0.002	*	*	*	*	*	*	*			*	*
10	10.076	0.320	0.002	*	*	*	*	*	*	*		*	*	*
11	12.000	0.311	0.002	*	*	*	*	*	*	*	*	*	*	*

Cp – Mallow Cp statistic, MSerr – Model Selection Error Rate

TABLE S4

A = Hypoxic Ventilatory Response
B = Hypoxic Burden (wake)
C = Arousal Threshold
D = Ventilatory Response to Arousal
E = Loop Gain
F = Apnea Duration
G = Arousal Index
H = Average SaO_2 during event
I = Apnea/Hypopnea Index
J = Body Mass Index
K = Age

Model	Cp	Adjusted R^2	MSerr	A	B	C	D	E	F	G	H	I	J	K
1	3.495	0.241	0.039	*										
2	1.395	0.269	0.038	*						*				
3	-0.647	0.297	0.037	*				*		*				
4	0.292	0.298	0.036	*	*			*		*				
5	0.622	0.305	0.036	*	*		*	*		*				
6	2.447	0.298	0.037	*	*	*	*	*		*				
7	4.214	0.291	0.037	*	*	*	*	*		*				*
8	6.094	0.283	0.037	*	*	*	*	*	*	*				*
9	8.011	0.274	0.038	*	*	*	*	*	*	*			*	*
10	10.001	0.264	0.038	*	*	*	*	*	*	*		*	*	*
11	12.000	0.255	0.038	*	*	*	*	*	*	*	*	*	*	*

Cp – Mallow Cp statistic, MSerr – Model Selection Error Rate
